# Studies of Olfactory System Neural Plasticity: The Contribution of the Unilateral Naris Occlusion Technique

**DOI:** 10.1155/2012/351752

**Published:** 2012-05-28

**Authors:** David M. Coppola

**Affiliations:** Department of Biology, Randolph Macon College, Ashland, VA 23005, USA

## Abstract

Unilateral naris occlusion has long been the method of choice for effecting stimulus deprivation in studies of olfactory plasticity. A significant body of literature speaks to the myriad consequences of this manipulation on the ipsilateral olfactory pathway. Early experiments emphasized naris occlusion's deleterious and age-critical effects. More recent studies have focused on life-long vulnerability, particularly on neurogenesis, and compensatory responses to deprivation. Despite the abundance of empirical data, a theoretical framework in which to understand the many sequelae of naris occlusion on olfaction has been elusive. This paper focuses on recent data, new theories, and underappreciated caveats related to the use of this technique in studies of olfactory plasticity.

## 1. Introduction

**Figure 1:**
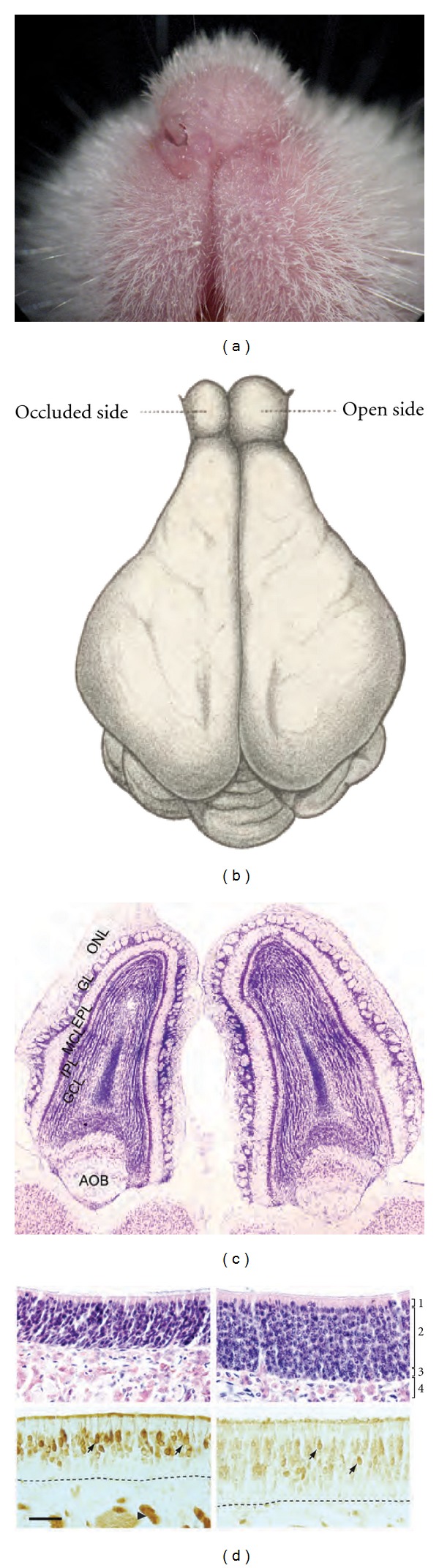
Anatomical and histological effects of UNO. (a) Adult mouse that underwent left UNO as a neonate. Note the normal right naris and apparently unaffected morphology around the location of the occluded naris. (b) Bernhard von Gudden's drawing of the brain from a young adult rabbit that had undergone UNO as a neonate ([4]; dorsal view reflected right/left to match other panels; occl: occluded). (c) Horizontal section through the olfactory bulbs of a young-adult mouse that had UNO as a neonate. Note all layers of the occluded bulb (left) are thinner than open-side bulb on the right (ONL: olfactory nerve layer; GL: glomerular layer; EPL: external plexiform layer; MCL: mitral cell layer; IPL: internal plexiform layer; GCL: granule cell layer; AOB: accessory olfactory bulb). (d) Histological sections through olfactory mucosa of young-adult mouse that had UNO as a neonate. Right column: open-side; left column: occluded side; top row: H&E stain; bottom row: OMP immunolabeling (arrows: mature OSN cell bodies) (layers: 1: sustentacular layer; 2: olfactory receptor cells; 3: basal cell layer; 4: lamina propria).

Ideas concerning the influence of deprivation and enrichment on the quality of human relationships can be summed up in the following aphorisms: concerning deprivation it is said that “absence makes the heart grow fonder,” a sentiment that has been expressed at least since Roman times; concerning abundance, Chaucer tells us that “familiarity breeds contempt,” a perhaps cynical but resonant view of human nature. Enlightenment thinkers, too, were keenly interested in the effects of deprivation and enrichment—not on the heart—but on the organ of thought. In 1688 the Irish politician and scientist William Molyneux posed a question, in a letter to John Locke, later known as “Molyneux's Problem,” concerning the role of experience in visual perception that was to occupy philosophers and scientists for the ensuing three centuries [1]. Later, Charles Darwin considered the heritable effects of deprivation and enrichment on the nervous system. He concluded in his 1868 opus entitled *The Variation of Animals and Plants Under Domestication,* after observing smaller crania in domesticated rabbits compared to their wild counterparts, “We thus see that the most important and complicated organ in the whole organization is subject to the law of decrease in size from disuse [2].” But it is the neuroanatomist and psychiatrist, Bernhard von Gudden, working at the same time as Darwin, who should be credited with pioneering the neurobiological study of sensory manipulation on brain development [3]. Among his other innovations, Gudden developed the unilateral deprivation model. Brilliant in its simplicity, this paradigm affords a within-subject comparison of different amounts of sensory stimulation on brain development. A century before Hubel and Wiesel won their 1981 Nobel Prize, in part, for their studies of the effects of unilateral deprivation by lid suture on visual cortex, Gudden had already invented the method and described its effects on the visual system in his monograph of 1870 [4, 5]. In this same series of studies he also described, for the first time, the effects of unilateral nostril occlusion (UNO) on the olfactory system, the topic of this paper. Gudden established that occluding one nostril of newborn rabbits caused a pronounced reduction in the size of the olfactory bulb on the same side after six weeks (Figure 1). While the history of UNO studies is neither as bulging nor ballyhooed as that of unilateral eyelid suture, the technique has, nevertheless, formed the mainstay of olfactory neuroplasticity studies and remains in active use more than four generations after its invention. In 1994 Brunjes provided an excellent review of the literature, up to that time, on the effects of UNO on the olfactory system [6]. Thus, after a brief discussion of olfaction and UNO phenomenology, the current paper will focus on more recent findings, new interpretations of the older literature, and remaining questions.

## 2. Basic Phenomenology

**Figure 2:**
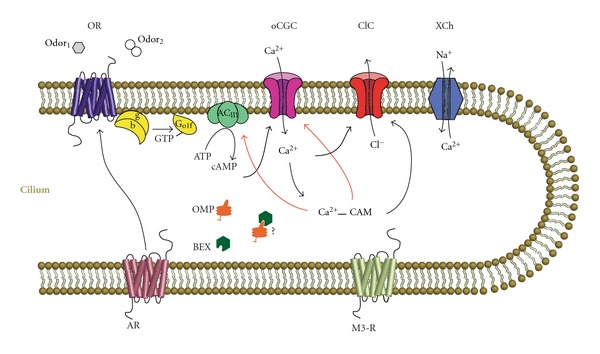
Simplified model of olfactory signal transduction within OSN cilium. AC⁡_III_: adenylyl cyclase; AR: adrenergic receptor; BEX: Brain-expressed X-linked protein; ClC: sodium/calcium exchanger; G_olf_: olfactory g-protein; M3-R: muscarinic acetylcholine receptor; oCGC: olfactory cyclic nucleotide-gated channel; OMP: olfactory marker protein; OR: odorant receptor. The Na^+^/K^+^/Cl^−^ cotransporter is not shown. Black arrows: stimulation; red arrows: inhibition (*after* [7] cf. [8]).

The mammalian olfactory system consists of an olfactory mucosa, sequestered in the dorsocaudal part of the nasal cavity, from which olfactory sensory neurons (OSNs) project their axons to the olfactory bulbs, rostral extensions of the telencephalon. At the bulbs, OSNs form synapses with output neurons, the mitral and tufted cells, in neuropil structures known as glomeruli. The largest known mammalian gene family codes for olfactory receptor (OR) proteins, a given OSN expressing but one of *∼*1000 genes in the mouse ([9]; Figure 2). All the OSNs expressing a given OR across the nasal cavity converge onto only a couple glomeruli, typically one medial and one lateral in each bulb. Odor information arriving at the bulb from the OSNs is processed by a highly laminar and complex set of direct and indirect pathways present in this well-studied structure [9]. Juxtaglomerular cells which, as their name implies, are part of the glomerular circuit and granule cells residing deeper are the key inhibitory interneurons of the bulb and are particularly important to our story. From the Mitral and Tufted cells olfactory information is transmitted to a group of central targets collectively known as the primary olfactory cortex including accessory olfactory nucleus, the piriform cortex, the entorhinal cortex, and the amygdala. It is in these central areas where a smell is given its appropriate perceptual and emotion qualities [9]. The following discussion will briefly consider the major developmental effects of UNO on each of the three tiers of the olfactory system starting with the bulb since this structure has received the most attention.

### 2.1. Olfactory Bulb

Gudden's principal discovery that UNO performed in the neonate causes the ipsilateral olfactory bulb to fail to reach its normal adult size has been replicated repeatedly in a number of different species (e.g., [10–13]; see Figure 1). Like Gudden, modern investigators have logically assumed that UNO's effects are due to odor deprivation to the occluded nasal fossa, though, as we will see, this assumption has only rarely been tested given the difficulty of olfactory deprivation by other means. The diminution of the ipsilateral olfactory bulb following UNO is due, in part, to reduction in the external plexiform and glomerular layers [14]. Indeed, the size of glomeruli, as judged in transgenic P2-receptor reporter mice, is smaller in the occluded bulb compared to the nonoccluded bulb within weeks of early postnatal occlusion [15–17]. However, the most dramatic decline after UNO in the ipsilateral bulb, by far, is in the granule cell layer ([14]; Figure 1).

 Earlier studies using tritiated thymidine and more recent studies using bromodeoxyuridine establish that the loss of granule cell layer volume from the occluded-side bulb is predominantly due to decreased cell survival not decreased neurogenesis [18, 19]. In contrast to these anatomical sequelae of UNO, which take weeks to detect, metabolic effects can be quite rapid. In the occluded-side olfactory bulb decreased 2-deoxyglucose uptake and Kreb-cycle enzyme immunochemistry is apparent in a matter of days after UNO [20, 21]. Also a rapid decline in protein synthesis, as measured by radiolabeled amino acid uptake, and change in gene expression, as measured by *in situ* hybridization, have been reported ([6, 22]; see the following).

Concerning bulb neurochemistry, an early observation was that tyrosine hydroxylase, the rate limiting step of dopamine synthesis, is markedly reduced in the ipsilateral bulb within days of nostril occlusion, an effect that can be reversed by reopening the nostril of experimental animals [23–25]. Tyrosine hydroxylase and dopamine content of juxtaglomerular cells, the predominant dopaminergic neurons of the bulb, decrease ipsilaterally after all of the following: UNO, olfactory nerve axotomy, or chemical lesion of the olfactory mucosa [24, 26, 27]. UNO causes a downregulation of *β*_1_ and *β*_2_-adrenergic receptors [28] but may have no effect on norepinephrine receptors [29]. Glutamate receptors, as a family, are not known to be effected by UNO but GluR1-positive short-axon cells are much reduced ipsilaterally in the external plexiform layer [30].

Neurotrophic factors, neuromodulators, and their receptors in the bulb are affected by UNO. Nerve growth factor receptors are increased on the occluded side 19 and 60 days after neonatal occlusion in the rat [31]. Brain-derived neurotrophic factor is initially increased and then later decreases ipsilateral to occlusion [32]. Insulin receptor kinase is downregulated on the occluded side; interesting since this receptor and its ligand are implicated in ion channel modulation as is BDNF [33–35]. The mitogen-activated protein kinase/extracellular signal-regulated kinase (MAPK/ERK) pathway, part of a key signaling-cascade, is also downregulated ipsilateral to occlusion [36].

 Some attention has been paid to the physiological or circuit effects of UNO on the bulb. In rats, three weeks of occlusion, starting on the day after birth, causes enhanced inhibition of mitral/tufted (M/T) cells in response to paired-pulse stimulation of the lateral olfactory tract [37, 38], an effect which appears to be NMDA receptor mediated [39]. In adult rats, either short-term (1-2 months) or long-term (12 months) UNO increased the proportion of M/T cells from the ipsilateral bulb that respond to multiple odorants suggesting a decrease in discrimination [40]. And in young rats even 15 min of naris occlusion causes a decoupling of M/T responses from respiration [41]. In a recent study, early UNO slowed the morphological development of ipsilateral mitral cells and checked the changes in membrane conductance and coupling coefficients that are part of the normal maturational shift from electrical to chemical synapses in the bulb [42]. Despite these results, electrophysiological studies have, for the most part, failed to show differences in the circuit properties of the ipsilateral bulb after UNO that are commensurate with the profound structural changes reviewed previously [43].

### 2.2. Mucosa

Studies of the effects of UNO, as in the case with visual and auditory deprivation, have tended to focus on central effects such that relatively less is known about the changes in the periphery resulting from this procedure. Metabolism, as measured by succinate dehydrogenase histochemistry, is measurably reduced in the ipsilateral mucosa of newborn rats within two days of occlusion [44]. In the mouse, rat, and rabbit, UNO leads to a substantial decrease in the thickness of the respiratory and olfactory mucosa ipsilaterally ([10, 45, 46]; see Figure 1). In the rat, early UNO causes a decline in the rate of mitosis in the olfactory epithelium [45, 47], a condition that can be reversed in a matter of days by reopening the closed naris [48]. Despite the difference in mucosal thickness, the number of mature olfactory sensory neurons is apparently unaffected by nostril occlusion in the mouse and rat ([10, 45] but see [15, 49]) though there appears to be a decrease in the rabbit, an inconsistency which has been attributed to the lack of a nasopharyngeal canal in the latter species [6, 46]. More recently, a histological study of rats occluded unilaterally from near birth to 60 days of age reported reorganization of tissue types in both the ipsilateral and contralateral mucosa compared to controls, most notably an expansion of olfactory mucosa on the occluded side [50]. In this vein, turbinate shape and positioning in three- to four-week-old mice, which had undergone UNO on the day after birth, are also affected such that the occluded-side turbinates take on a fine filigree-like appearance, especially rostrally, compared to their more robust partners on the open side [51]. Finally, a rapidly growing line of research is implicating OSN activity in the expression of axon guidance molecules. In these studies, UNO is often used to compare the abundance of guidance molecules in open and occluded-side mucosa as evidence for their activity dependence (e.g., [52–54]).

### 2.3. Central Pathways

The potentially important topic of the effects of early UNO on the development of central pathways has unfortunately garnered rather little attention judging by the literature. In one study, the thickness of piriform cortex layer 1b and the size of semilunar cell dendrites were reduced ipsilaterally in postnatal day (PND) 30 rats occluded on PND1 [55]. In a recent study, the expression of the NMDA receptor NR2B and the phosphorylated form of the regulator element CREB were downregulated in the piriform five days after naris occlusion, an effect which could be fully reversed ten days after reopening of the naris [56]. And early postnatal UNO in rats delays the normal developmental increase in the ratio of AMPA receptors to NMDA receptors at primary sensory synapses but not associational synapses on pyramidal neurons in piriform cortex slice preparations [57]. In a previous related study, field potential recordings from intact anterior piriform cortex establish an ipsilateral depression of responses evoked by stimulation of cortical afferents in early (PND1) but not late (PND 30) occlusion [58]. However, in this study evoked potentials in intracortical association fiber were enhanced ipsilaterally in both early and late-onset UNO rats.

Concerning other central olfactory structures, UNO from PND 1–20 caused a decrease in 2-deoxyglucose uptake in the rostral anterior olfactory nucleus [59]. However, published reports on the effects of UNO on other central olfactory structures such as the amygdala and entorhinal cortex are lacking.

 Collectively, these rather modest effects ipsilateral to UNO in higher brain centers have been attributed to the bilateral inputs of these structures [6]. Nevertheless, given the current paucity of studies more work in this area would be of benefit.

### 2.4. Human Studies

Olfactory bulb size measured by MRI has been positively correlated with olfactory psychophysical test scores in clinical populations recovering from head-trauma as well as in normal adults and in young people [60–62]. That this relationship is causally linked, and more importantly, plastic over the lifetime, is suggested by research showing that patients with the most severe chronic rhinosinusitis (CRS) tended to have the smallest olfactory bulb volumes and poorest olfactory performance [63]. Moreover, a longitudinal study of CRS patients under a standard treatment regimen showed an increase in bulb size accompanied by a decrease in odor thresholds after treatment [64]. Together, these clinical findings suggest that levels of peripheral input in humans may affect cell survival in the olfactory bulb and other olfactory processes as they do in rodents (see the following). More to the point, there has been at least one study of short-term (one-week) UNO in humans accompanied by a similar duration recovery period [65]. Psychophysical testing and fMRI analysis of subjects after deprivation and again after recovery provide modest evidence that odor deprivation induced a reversible *increase* in odor detection with a concomitant decrease in the specificity of odor coding in the piriform cortex.

## 3. A Method in Search of a Theory

While admittedly the aforementioned literature review focuses on positive results, it would appear that wherever one looks, and whatever the endpoint—anatomical, biochemical, or physiological—UNO has marked effects on the ipsilateral olfactory system. However, a theoretical framework in which to place the myriad effects of naris occlusion has been more elusive. One obvious solution, contemplated by Meisami [12] upon his modern rediscovery of Gudden's method, was to place these findings in the now massive corpus establishing the indispensable role of activity in normal neural development [66, 67]. In this formulation, neural activity is believed, through Hebbian mechanisms, to strengthen appropriate functional connections and weaken, ultimately weeding out, inappropriate connections [6]. It is perhaps ironic that arguably the best understood example of this process comes from studies of the effects of monocular lid suture (Gudden's technique) on ocular dominance column formation in the visual cortex [68]. But there are reasons to question whether Hebb's postulate and the unavoidable comparisons to ocular dominance plasticity are really appropriate to UNO phenomenology. For example, the notion that sensory experience might play an instructive role in the layout of the olfactory map in the bulb has collapsed under the weight of contrary evidence. Modern genetic approaches, which have allowed the creation of mouse strains lacking essential components of the transductory cascade, establish that the proper guidance of sensory cell axons to bulbar targets does not require sensory activity or even functional synaptic release, though spontaneous activity (i.e., nonsensory driven and presumably uncorrelated) seems to be necessary ([69] reviewed in [9]). Given the problem that developmental linguists have colorfully referred to as the “poverty of the stimulus”, it was probably never a tenable proposition that >1000 types of OSNs could sort their axons in a matter of a few days of development, based on sensory-driven correlated activity [69]. Thus, Hebb's postulate finds little succor in what we have recently learned about the development of the bulb odor map; however, intrabulbar connections may be a different matter [15]. But there are additional problems to consider with the analogies between experience-dependent plasticity in other sensory systems and the effects of UNO on the olfactory system.

### 3.1. Critical Period

One line of evidence in favor of the paradigm described previously comes from early evidence that the effects of UNO have a “critical period” like the sharply defined boundaries of ocular dominance plasticity. In rats, it was shown that UNO from PND 1–30 caused the typical 25% reduction in ipsilateral bulb size, while occlusion from PND 30–60 or PND 60–90 had little effect [70]. However, in subsequent studies many of the effects on the ipsilateral bulb after early postnatal UNO have been shown to accrue after adult occlusion including reductions in size [71–73], neurogenesis (e.g., [72]), and granule cell branching [74]. Adult UNO also causes a rapid bilateral increase in cell death within layer I/II of the piriform [75]. Moreover, unlike the case for ocular dominance column plasticity, UNO effects are reversible, for an apparently indefinite period, upon naris reopening [76]. Thus, the bulk of the available data does not support a “critical” or sensitive period for UNO effects on the olfactory bulb.

### 3.2. Firing and Wiring

At an even more fundamental level, however, the analogy between activity-dependent processes in other sensory systems and the phenomenology of UNO seem inapt, particularly concerning its effects on the bulb. The profound reduction in bulb size that accompanies UNO has no obvious precedent in other sensory systems deprived of their appropriate stimulus. In the visual system, even total stimulus deprivation by dark-rearing does not cause anything like the size change and cell loss seen in the olfactory bulb after UNO [68]. For example, in the retina, the structure in the visual pathway perhaps most analogous to the olfactory bulb, dark-rearing causes a decreased pruning of retinal ganglion cell dendrites not a net loss of cells [77]. Even the celebrated decrease in deprived-eye ocular dominance columns in visual cortex after monocular deprivation is due to the competitive environment of in-growing binocular inputs [68]. No analogous competition exists in the exclusively unilateral afferents to the olfactory bulb. Indeed, dark-rearing has rather modest effects on the size and wiring of visual pathways provided that it does not persist too long into young adulthood [60]. Importantly, it is the *pattern* not the total amount of activity that seems to be the critical determinant of experience-dependent plasticity in the visual system [68, 77]. These aspects of the effects of stimulus deprivation on the visual system also appear to apply to the somatosensory system [77].

### 3.3. Persistent Neurogenesis

Admitting that the effects of UNO on the olfactory bulb are unique among sensory systems, one has to seek unique explanations. A singular and striking feature of the olfactory system is its continuous turnover of OSNs and supply of new interneurons to the olfactory bulb from the rostral migratory stream (RMS).

In the olfactory mucosa precursor cells near the basal lamina (Figure 1) divide to become new OSNs sending dendrites toward the mucosal surface and axons that make their way to the olfactory bulb where they gain functional connection in glomeruli (reviewed by [78]). This process, which occurs throughout life, underlies the replacement of dying mature neurons in a cycle with a period of a few months [78].

The bulb's continuous supply of new RMS neurons differentiates predominantly into juxtaglomerular cells and granule cells, both inhibitory interneurons. A spate of recent studies establishes that some of these adult-born neurons survive and become functionally integrated in an activity-dependent manner (reviewed in [79, 80]). As noted previously, UNO decreases the survival of adult-born neurons in the bulb and, amazingly, olfactory “enrichment” increases their survival [72, 80]. Behavioral studies suggest that the incorporation of adult-born neurons into the olfactory bulb may actually play important functional roles in certain types of olfactory learning and memory [80]. While a complete review of the burgeoning literature on olfactory bulb adult-born neurons is beyond the scope of this paper, it is mentioned here as a potential explanation for the dramatic effects of UNO on bulb size and other parameter. Given that most of the decrease in ipsilateral bulb size after UNO is related to granule cells loss and stimulus-driven activity is necessary for granule cell survival, it follows that UNO, which surely reduces stimulus driven activity, would lead to a decline in bulb size in adults and failure to reach full size in young animals. Of course this analysis merely begs the question of why the visual, auditory, and somatosensory systems manage to function efficiently without ongoing neurogenesis. One suggestion is that bulbar neurogenesis can be understood in the context of a neural circuit in which the inputs—the OSNs—are undergoing continuous turnover for the purpose of adjusting to an ever-changing odor environment [80]. An important caveat to this line of reasoning emerges from recent studies of the RMS in humans [81]. While infants less than 18 months have an extensive population of newborn migrating neurons forming a substantial RMS, this germinal activity subsides thereafter and is virtually nil in adulthood. At least on its face, this result seems at odds with the view that adult-born neurons from the RMS play an essential role in ongoing olfactory function, especially given the very respectable olfactory capabilities in the average adult human [82].

 Apart from the recent human data, the literature on olfactory neurogenesis is perplexing. As we have seen UNO causes a reduction in neurogenesis in the olfactory mucosa of young and adult animals and a decrease in granule cell survival in the olfactory bulb [45, 47, 72]. Interestingly, UNO also causes decreased neurogenesis within the nonsensory respiratory epithelium leading to the suggestion that something in respired air other than odors, such as toxins, microorganisms, or other foreign particles, might be driving mitogenesis [45]. Evidence in support of this idea came from an early study showing that many OSNs could live for up to a year in mice (the species' entire normal life expectancy) provided that the animals were raised in a laminar flow hood to prevent nasal infections [83]. Add to these findings a recent study showing that odor exposure, in bulbectomized mice, rescues OSNs from the apoptosis that usually accompanies this manipulation [84]. Whether odors or other factors in respired air govern the death and replacement of OSN in the olfactory mucosa and how this, in turn, effects death and replacement of interneurons in the bulb remain open questions, as does the evolutionary significance of this unprecedented plasticity.

 Thus, it must be concluded that, despite the existence of so many excellent empirical studies, a satisfactory theoretical framework in which to understand the effects of UNO on the olfactory system has eluded us so far!

## 4. Problematic Paradigm

The cliché “nothing succeeds like success” surely applies to the UNO technique. As already noted, the number of sequelae of this manipulation within the olfactory system is large and growing. It remains the method of choice for stimulus deprivation available to any investigator with a cautery. However, the assumptions underlying its application have rarely been tested.

### 4.1. Deprivation

Unlike the case with dark-rearing or lid-suture (contrast deprivation) in vision studies, nothing like complete deprivation is achieved by UNO. Electrophysiological recordings in rats [41] and Fos immunohistochemistry in ferrets [85] reveal odor-driven activity in the ipsilateral bulb even after acute UNO. Using either acute or long-term UNO paradigms, rats that have undergone contralateral bulbectomy can detect odors at extremely low concentrations [86, 87]. And similarly prepared newborn mice can use odor cues to find their mother's nipple and navigate back to the nest [88]. Finally, adult mice that received UNO as newborns and contralateral bulbectomy as adults can perform *better* than controls (unilateral bulbectomy *without* contralateral UNO) in both odor habituation/cross-habituation and operant testing ([89]; see the following). One explanation for these results is that in rodents and some other mammals there is a surprisingly large nasopharyngeal canal that could allow odor mixing between the open and occluded nasal fossa [86]. Also, odors undoubtedly gain access to the occluded fossa by a retronasal route. Indeed the negative pressure that must be created in the occluded fossa with each inhalation *guarantees* a substantial exchange of air between the occluded fossa and the outside world by internasal and retronasal passage.

 One last point on the deprivation achieved by UNO concerns the question of *what* the nasal cavity is actually being deprived of. The profound and comprehensive effect that UNO has on the interneuron population of the ipsilateral bulb stands in stark contrast to the subtotal, potentially regional, and environmentally dependent deprivation that occurs upon occluding a naris. Already noted are the substantially preserved olfactory capabilities of rodents forced to smell with only their occluded-side olfactory system intact, a feat requiring the rerouting of odor entry to the nasal fault. These considerations suggest that the interneuron population in some regions of the olfactory bulb should be spared by UNO. Even more fundamentally, the odor environment of the average laboratory or animal facility must be impoverished compared to a natural environment. Given this situation it seems likely that most of the 1000s of different types of OSNs (based on the olfactory receptor they express) are deprived of their specific ligand most of the time in the laboratory environment. In this light, the global effects of UNO on the bulb are all the more surprising. Is not deprivation in an impoverished environment of less moment than deprivation in an enriched environment? Considering these facts it is interesting that OSNs, in addition to responding to odor ligands, are exquisite mechanoreceptors [90]. There can be little doubt that UNO causes marked and global decreases in mechanical force in the occluded fossa that would normally accompany respiratory airflow. Thus, it could be speculated that mechanical force deprivation may explain the global effects of UNO on the bulb provided that one also posits a role for OSN mechanical transduction in this activity dependence process.

 In search of an effect of nostril occlusion commensurate with the global effects on the interneuron population of the bulb it is worth noting, as mentioned previously, that other factors in air: irritants, microbes, toxic substances, and the like, that the occluded-side nasal fossa is partially protected from, should be given more attention as potential causes of mucosal and bulbar changes following UNO. It is relevant in this regard that trigeminal sensory fibers richly innervate the olfactory mucosa sending collaterals to the olfactory bulb where they are thought to have a modulator influence [91]. Given the existence of this circuit, it seems possible that some of the effects of UNO on the ipsilateral bulb may be related to interference with the normal interplay between the trigeminal and olfactory systems.

### 4.2. Specificity

**Figure 3:**
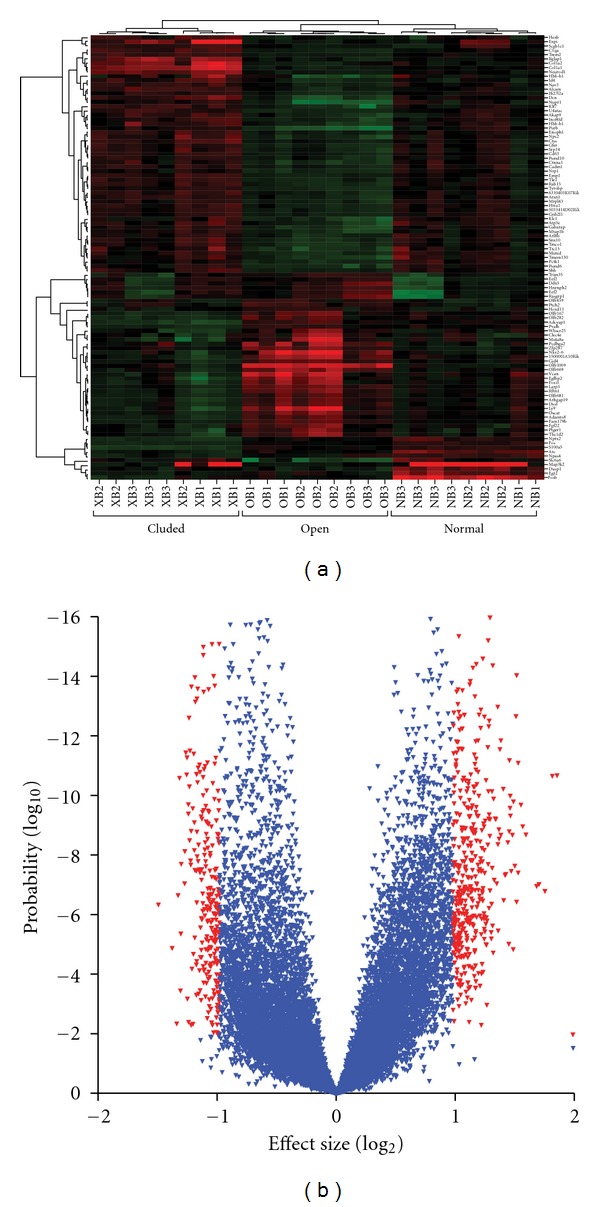
Microarray analysis of the effects of early postnatal UNO on olfactory bulb transcriptome of young adult mice. (a) Expression profile of 103 genes from the >20,000 total on the chip that met arbitrary significance criteria (2.25-fold up, or 2.25-fold down, with *P* < 0.01). Tissue source is shown on bottom axis. Note that there were three technical replicates within each of three biological replicates (subscript numbers). Color represents expression value with red, upregulation and green, downregulation. Dendrograms based on expression values show clustering genes (left) and samples (right), respectively. Note large number of up- and downregulated genes in both occluded and open (nonoccluded) bulb with normal bulb showing intermediate expression of most genes. (b) Volcano plot of 16,456 genes detected by the array for the comparison of occluded versus open olfactory bulb in UNO mice. Transcript abundance (log_2_) is plotted on the abscissa. Statistical significance (log_10_) is plotted on the ordinate. Genes shown in red meet a 2-fold and *P* < 0.01 criterion. Note that there are more upregulated genes (+) than downregulated genes (−) on occluded side.

Another implicit assumption of the UNO technique is that its effects are systemically benign and limited to olfaction. However, investigators have repeatedly shown that animals grow at a slower rate after UNO compared to controls (e.g., [12, 88]). In contrast to deprivation directed at the eye or the ear, the nasal cavity has a number of functions besides olfaction not least respiration. Local reductions in oxygen, increases in carbon dioxide, and the aforementioned protection from drying, irritants, and microbes could all be factors underlying some of the effects of UNO. To this point and as noted previously, turbinate morphology is abnormal on the occluded side of young adult mice after early postnatal UNO, an effect unlikely to be related to odor deprivation [51]. Finally, we have recently compared the transcriptomes of ipsilateral and contralateral UNO mice to those of untreated mice ([92]; Figure 3). A number of genes, seemingly unrelated to olfaction, are regulated in the occluded-side mucosa and bulb, casting further doubt on the specificity assumption.

### 4.3. Contralateral Control

One of the most egregious suspensions of the basic tenants of experimental design occurs routinely in experiments using the UNO technique. Perhaps because some of the early investigators, dating back to Gudden, assumed that the contralateral side was “normal” and if true that this would afford a powerful within-subjects experimental design, many modern UNO aficionados use few or no control subjects in their studies. Yet, common sense and experimental evidence suggest that the contralateral side of UNO subjects is not normal. While on the occluded side the airflow is dramatically reduced, especially rostral to the nasopharyngeal canal, the open side is forced to carry a larger-than-normal volume of air (presumably twice the amount). Also, UNO abrogates alternating cycles of breathing, forcing constant duty on the open side. Not surprisingly this leads to detectable histological and physiological changes in the contralateral mucosa. Most dramatic is the profound loss of OSNs in the rostral end of the nasal cavity after long-term (≥ six weeks) UNO in mice [93, 94]. Similarly, a suite of histological abnormalities has been noted in the contralateral mucosa of rabbits [95] and rats [50] after UNO. In mice, there is hypertrophy of turbinates from the contralateral nasal fossa compared to those from untreated subjects, within 18 days of early postnatal UNO [51]. Finally, one finds several differentially regulated genes comparing the transcriptomes of control and contralateral mucosa and bulb in young-adult mice that received UNO as newborns ([92]; Figure 3(a)). Some of these genes are not yet annotated, and others seemingly are unrelated to olfaction; however, they stand as existential proof that the contralateral side of UNO subjects is not “normal.”

### 4.4. Compensatory Processes

In contrast to the Hebbian view of olfactory development discussed previously, evidence has recently accumulated for the opposite proposition. Indeed many of the changes in the olfactory system following UNO appear to be compensatory in that they cause changes in the system that work to preserve olfactory function in the face of sensory deprivation [96]. For example, olfactory bulb neurotransmitter systems seem to follow this pattern in that the decrease in ipsilateral bulb dopamine following UNO is compensated for by a >30% increase in dopamine D_2_ receptors that cannot be ascribed to shrinkage of lamina [97]. Analogously, the increase in ipsilateral bulb norepinephrine is compensated, in part, by a decrease in norepinephrine receptors [96]. Also, while UNO may cause an ipsilateral decrease in the extent of glomerular neuropil and the dendritic arbor of mitral cells, it also causes a more uniform distribution of a synaptic protein synaptophysin, a response that may be viewed as compensatory [96]. Finally, in a recent study, the depletion of ipsilateral granule cells following UNO appears to be compensated for by an increased excitability among the remnant granule cell population [19]. All of these examples may help explain how the ipsilateral olfactory bulb of animals subjected to long-term UNO appear to function normally, as far as we know, despite its abnormal morphology.

 Evidence of compensation also abounds at the first synapses of the olfactory system and in the periphery. Tyler et al. published among the first detailed studies of the effects of UNO on primary and secondary synapses in the olfactory system [98]. Using the whole-cell voltage-clamp technique in a rat slice preparation, they showed that two weeks of olfactory deprivation, beginning on PND2, increases the probability and quantal content of neurotransmitter release at primary olfactory synapses in the ipsilateral bulb. This effect of UNO could be demonstrated as early as three days after the onset of naris occlusion in young adult rats. Furthermore, immunolabeling of the vesicular glutamate transporter and two glutamate receptor subunits demonstrated that UNO caused an upregulation of these components at ipsilateral primary olfactory synapses. Voltage-clamp recordings of spontaneous and olfactory-nerve-evoked activity in the predominant second-order neurons of the bulb, including mitral cells, revealed that UNO also strengthens synapses in down-stream components of the olfactory circuit. This latter finding may explain earlier observations that the size and intensity of odor-induced 2-deoxyglucose foci are increased in the ipsilateral-bulb glomerular-layer of UNO rats after reopening the occluded naris [43]. In this earlier study it was observed that more ipsilateral than contralateral mitral cells respond to a given odorant. Collectively, these studies reveal a previously unknown compensatory response, namely, that primary and secondary olfactory synapses are strengthened ipsilaterally after UNO. Such strengthening of primary and secondary synapses following deprivation is also hard to square with a Hebbian process being more consistent with the notion of homeostatic plasticity [98, 99].

 What about the most peripheral components of olfaction—the OSNs? One clue that compensatory processes may exist in these sentinels of smell came from the still unexplained observation that olfactory marker protein (OMP) immunolabeling is more intense in the ipsilateral than contralateral (or control) mucosa of mice subjected to UNO ([100]; Figure 1(d)). Given that *less* stimulation led to *more* OMP in this study, some sort of compensatory process was suggested, especially given this protein's suspected function in olfactory transduction [101]. In a series of follow-up experiments, it was shown that adenylate cyclase type III (AC⁡_III_), a major component of the olfactory transductory cascade, and a nonciliary phosphodiesterase, which has been shown to be involved in transductory modulation, were also upregulated in OSNs in response to nostril occlusion [102]. At least for the AC⁡_III_ result the implication was clear: decreased olfactory stimulation leads to an increase in this enzyme whose product cAMP ultimately causes OSNs to reach threshold for action potential initiation. Thus, stimulus deprivation could be setting in motion a biochemical cascade leading to an increase in “gain” in the OSN transductory cascade (Figure 2). Microarray analysis has recently been used to confirm and extend the previously findings based on immunolabeling [92]. The transcriptomes of adult olfactory mucosa from control mice were compared to those from the ipsilateral and contralateral sides of mice subjected to UNO as neonates. Transcripts of key genes involved in olfactory reception, transduction, and transmission including many olfactory receptors, the olfactory G-protein, the olfactory cyclic nucleotide gated channel, the olfactory calcium-activated chloride channel, and AC⁡_III_, were upregulated in deprived-side olfactory mucosa, with opposite effects in nondeprived-side mucosa, compared to controls. Thus, these microarray results support the hypothesis that the odor environment can trigger a previously unknown homeostatic control mechanism in OSNs.

 Of course, if these observations at the gene and protein level have any functional significance, they should be measurable electrophysiologically. To address this issue EOG recordings were collected from matched locations on the olfactory mucosa from the ipsilateral and contralateral nasal cavity of UNO mice [103]. The stimulus set included a log-dilutions series for a number of odorants common in olfactory research. Consistent with the gene and protein data, EOG amplitudes from recording sites on the deprived mucosa of UNO mice were greater for a given odorant and concentration of stimulus than those from the open side. For some subjects the magnitude of the EOG on the occluded side was as much as double that on the open side. Given that the EOG is thought to be derived from the summed generator potential of OSNs in the vicinity of the recording electrode, these results imply that OSNs on the occluded side have larger generator potentials or that more cells are recruited by a given odor, or both. These electrophysiological results, as for the protein and RNA data, are consistent with the hypothesis that OSNs respond to deprivation in a compensatory manner. This process would seemingly oppose any Hebbian pruning of synaptic connections in the bulb.

 However, the fact still remains that UNO causes reduction in the survival of proliferating granule cells and other interneurons in the bulb [19, 30]! Given that granule cells are inhibitory on mitral cells, the major output neurons in the bulb, and are thought to participate in lateral inhibition that may sharpen odor discrimination, it is tempting to suggest that a homeostatic process in the olfactory circuit underlies their loss after UNO. Whatever else the function of the persistent supply of granule cells to the bulb (see the aforementioned part) their decline with deprivation would allow the system to increase odor detection, perhaps at the price of odor discrimination [40]. It is interesting in light of these considerations that mice whose bulbs are infused with a drug that limits neurogenesis have surprisingly normal olfactory capabilities [104]. Moreover, as noted previously, mice forced to rely only on their occluded olfactory system by removing their contralateral bulb perform better in behavioral tests of detection than control mice [89].

## 5. Residual Puzzles

Some of the outwardly conflicting results of UNO on the olfactory system discussed so far in this paper may not turn out to be incompatible. Hopefully additional research will make clear what now seems inconsistent. Nevertheless, there may be worth noting some additional lines of evidence concerning the effects of odor experience on the olfactory system that must be considered in any comprehensive theory of olfactory plasticity.

### 5.1. Olfactory Induction

The specific anosmia to androstenone, which occurs in roughly half of the human population, can be reversed upon repeated exposure to this odorous steroid [105]. This result has been replicated in certain strains of mice, as has induced sensitivity to certain other odorants [106]. Based on EOG recordings, the locus of this effect has been shown to be the olfactory mucosa, at least in part [106, 107]. Consistent with these results, rats trained in an odorant detection task showed heightened responses and altered mucosal response patterns to the trained odors compared to those in age-matched controls [108]. Perhaps most surprisingly such olfactory induction can occur trans-utero, as rabbits whose mothers have been fed juniper berries show heighted EOG responses to juniper odor postnatally [109]. No mechanism for olfactory induction has been established but the previously discussed evidence that odor exposure, in bulbectomized mice, rescues OSNs from the apoptosis that usually accompanies this manipulation may be pertinent [84]. Perhaps odor exposure changes the population make-up of OSN types in the olfactory mucosa.

### 5.2. Olfactory Perceptual Learning

This is a phenomenon that in some ways is reminiscent of olfactory induction (reviewed by [110]). After a period of passive exposure to certain binary mixtures of pure odorants, rats begin to discriminate components that had not been discriminated prior to the exposure period [111]. However, unlike induction, this phenomenon is thought to have a bulbar origin because blocking neurogenesis in the bulb with drug infusion before and during the odorant exposure period prevents the improvement in discrimination [111].

### 5.3. Compensation Redux

From an evolutionary perspective the compensatory processes discussed previously, which appear to be implemented at various levels of the olfactory system, seem quite logical. Given their finite dynamic range, nature has designed sufficient plasticity into sensory systems to continuously adjust their output to maximize the useful information transferred by them about the environment to the brain [112]. This is why sensory systems modulate in order to report *changes* in the environment rather than static levels of a stimulus [113]. Adaptation is a short-term example of this mechanism that has been examined extensively, both empirically and theoretically, in many sensory systems (e.g., [114]). The effects of longer-term deprivation on the olfactory system, such as those seen following UNO, can be understood in the same light as adaptation, though their cellular mechanisms, time course, and reversibility may be quite different. From this viewpoint, animals exposed to “noisy” or “enriched” odor environments might be expected to show changes opposite to those reported for the deprived state. This is exactly the kind of push-pull mechanism that was seen in the microarray studies discussed previously, with control transcript levels intermediate between ipsilateral and contralateral values for the most important olfactory transductory elements [92]. Consistent with such a compensation mechanism, a recent study has shown that transgenic mice with a gene-targeted potassium-channel deletion that renders mitral cells hyperexcitable actually lose many OSNs [93].

Olfactory induction and perceptual learning are more difficult to understand from an ethological viewpoint. Absent any behavioral relevance, why should the nervous system ramp up detection and discrimination of odors that it is merely exposed to that may have no survival value whatsoever? In any event, the empirical results showing that UNO causes upregulation of olfactory transductory elements ipsilaterally (deprived) and downregulation contralaterally (enriched) appear, at least on their face, to be at odds with the prediction of olfactory induction and perceptual learning. The preserved olfactory competence measured at the behavioral level seen in animals with long-term UNO coupled with contralateral bulb ablation also seems inconsistent with these predictions [89]. Notably, human subjects that are chronically exposed to a particular odor for a few weeks show *increases* in threshold that are odor specific, another finding that seems incompatible with the phenomena of induction and olfactory perceptual learning but is completely compatible with the predictions of compensation [115].

**Figure 4:**
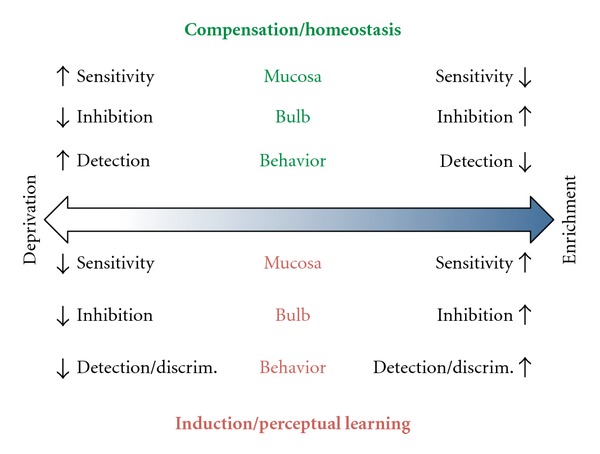
Diagram of the predicted effects of odor deprivation (left) and enrichment (right) according to the compensation/homeostasis hypothesis and induction/perceptual learning hypothesis on the olfactory mucosa, bulb, and behavior. Compensation makes no specific prediction about odor discrimination but this may necessarily be opposite to the predicted effects on detection based on bulbar neural circuits. Induction predicts greater detection under enrichment. Perceptual learning predicts greater discrimination under enrichment (see text for further explanation).

 As a heuristic exercise, the predicted effects of deprivation or enrichment on the olfactory system at the levels of the mucosa, bulb, and behavior can be contrasted for the induction, perceptual learning, and compensation paradigms (Figure 4). Notably, in some circumstances the predicted effects of these processes are congruent and in others they are in conflict.

## 6. Conclusions

Gudden, mentor of Emil Kraepelin and Franz Nissl among other notables, was arguably the very first neurobiologist. He pioneered the technique, which still bares his name, of using secondary degeneration to study interrelationships between cortical and subcortical structures and as a psychiatrist he helped humanize the treatment of the insane [3]. Ironically, he most certainly died at the hands of his psychiatric patient “mad King Ludgwig II” of Bavaria, though the details of their simultaneous drowning are shrouded in mystery. It is interesting to ponder what Gudden would think of the progress that has been made in the intervening century since he invented his unilateral deprivation techniques. He might not be surprised to find out that the role sensory activity plays in the development and maintenance of the nervous system turns out to be an incredibly intractable problem. For example, some complex computed sensory modules, like orientation maps in visual cortex, develop normally without the benefit of sensory input, while other modules that coexist in the same cortical volume, such as visual direction domains, have an absolute requirement for visual experience [104, 116, 117].

 The UNO technique will undoubtedly remain an indispensible tool in the armamentarium of olfactory neuroscientists despite the shortcomings of the procedure and the conflicting results and hypotheses it has engendered. Key among the matters remaining to be resolved is the correct theoretical framework in which to understand the effects of deprivation on olfaction. Romantics will continue to debate the influence of a lover's presence or absence on the ardor of the human heart. Likewise for the olfactory system we would like to know if absence make the nose grow fonder? Or is it a case of out of smell, out of mind?
